# Identification of tick-borne pathogen diversity by metagenomic analysis in *Haemaphysalis longicornis* from Xinyang, China

**DOI:** 10.1186/s40249-018-0417-4

**Published:** 2018-05-07

**Authors:** Lu Zhuang, Juan Du, Xiao-Ming Cui, Hao Li, Fang Tang, Pan-He Zhang, Jian-Gong Hu, Yi-Gang Tong, Zhi-Chun Feng, Wei Liu

**Affiliations:** 1 State Key Laboratory of Pathogen and Biosecurity, Beijing Institute of Microbiology and Epidemiology, 20 Dong-Da Street, Fengtai District, Beijing, 100071 People’s Republic of China; 2 Affiliated Bayi Children’s Hospital, PLA Army General Hospital, 5 Nan-Men-Cang, Dongcheng District, Beijing, 100700 People’s Republic of China; 3 National Engineering Laboratory for Birth Defects Prevention and Control of Key Technology, 5 Nan-Men-Cang, Dongcheng District, Beijing, 100700 People’s Republic of China; 4 Center for Diseases Control and Prevention of Chinese Peoples’ Armed Police Forces, Beijing, 102613 China

**Keywords:** *Haemaphysalis longicornis*, Pathogens, High-throughput sequencing

## Abstract

**Background:**

A wide variety of pathogens could be maintained and transmitted by *Haemaphysalis longicornis*. The aim of this study is to systematically examine the variety of pathogens carried by *Haemaphysalis longicornis*, an importnatn vector, in tick-borne diseases epidemic area, and to estimate the risk of human infection imposed by tick bites.

**Methods:**

Adult questing ticks were collected in Xinyang, central China. Genomic DNA and RNA were extracted from 144 *H. longicornis* ticks individually, and sequenced respectively as the templates for high-throughput sequencing. Clean reads were compared against the database of NCBI nucleotide collection and specific PCR was performed to confirm the presence of pathogen. Phylogenetic analysis was performed to explore the evolutionary status of pathogens.

**Results:**

The assignment of reads to taxa based on BLASTN results revealed the existence of several potential pathogens, including *Anaplasma* spp., *Rickettsia* spp., *Babesia* sp., as well as severe fever with thrombocytopenia syndrome bunyavirus (SFTSV). Comfirmantory PCR assays revealed the existence of *Anaplasma bovis* (13/144, 9.03%), *Anaplasma centrale* (2/144, 1.39%), *Rickettsia heilongjiangensis* (3/144, 2.08%), *Rickettsia* sp. LON-13 (1/144, 0.69%), *Rickettsia raoultii* (5/144, 3.47%), *Babesia* sp. (1/144, 0.69%). SFTSV accounted for the highest detected pathogen with a positive rate of 18.75% (27/144). Three of the ticks (2.08%) were co-infected with SFTSV and *A. bovis.*

**Conclusion:**

Our study provided a broadened list of microorganism that harbored by *H. longicornis*. In previously unrecognized endemic regions, prokaryotic and eukaryotic infection including *Anaplasma* spp*.*, *Rickettsiae* spp., and *Babesia* spp*.* should be considered, along with the well-known SFTSV for patients with tick bites history. A novel *Babesia* species was identified in local natural foci, which needs further investigation in the future.

**Electronic supplementary material:**

The online version of this article (10.1186/s40249-018-0417-4) contains supplementary material, which is available to authorized users.

## Multilingual abstracts

Please see Additional file 1 for translations of the abstract into the five official working languages of the United Nations.

## Background

*Haemaphysalis longicornis* is one of the most important tick species that imposes the risks of tick-bonre disease infection, which is widely distributed in the Asia-Pacific region, including Korea, Japan, Australia, the Pacific Islands, and New Zealand [1–5]. Common hosts of *H. longicornis* include goats, cattle, sheep, *Bos mutus*, donkeys, pigs, *Cervus elaphus*, cats, *Rattus norregicus*, *Mus culus*, *Erinaceus europaeus*, *Mustela sibirica*, *Trichosurus vulpecula*, and some birds, along with human beings, which is commonly considered as the definitive host [5–7]. A wide variety of pathogens can be maintained and transmitted by *H.longicornis*. A remarkable example is the novel phlebovirus in the Bunyaviridae family (severe fever with thrombocytopenia syndrome virus, SFTSV), which was newly identified to be a causal agent of severe fever with thrombocytopenia syndrome (SFTS) in China and other neighbouring countries [8–10]. SFTS is largely considered to be a tick-associated disease, as high proportion of patients had tick exposure before disease onset [8]. *H. longicornis*, the most prevelant tick species that infests human in SFTS-endemic areas, was determined to be a competent vector of SFTSV by an experimental maintenance and transmission study [11]. In this context, we performed a metagenomic analysis to provide an inventory of predicted and unexpected pathogenic agents carried by *H. longicornis* ticks, captured in Xinyang Administrative Area, Henan Province in central China.

Xinyang is the region mostly heavily inflicted by SFTSV, reporting 48% of SFTS cases in China [12]. The region has a humid subtropical climate with annual precipitation of around 1100 mm. The southern part of Xinyang, stretching across the Dabie Mountain range, is an important habitat for *H. longicornis* ticks in China [13]. Xinyang is also reported to be epidemic area of many tick-borne diseases such as human granulocytic anaplasmosis, spotted fever and typhus fever [14, 15]. These findings indicated that *H. longicornis* might be potential vector of causative agents. Therefore we expect the metagenomic analysis in this region might provide a broad list of the pathogens carried by this important vector, thereby offering the potential humans infection risk imposed by tick bites.

## Methods

### Ticks collection and DNA/RNA extraction

The questing ticks were captured from 10 sampling sites across Xinyang. Sample sites were selected from representative geographical areas where patients got tick bite in Dabie Mountain area and plain area. (Dabie Mountain area: 31.69 N, 115.45E; 31.59 N, 115.30E; 31.76 N, 115.27E; 31.70 N, 114.82E; 31.66 N, 115.01E; plain: 32.48 N, 115,31E; 32.40 N, 115.24E; 32.38 N, 115.45E; 32.25 N, 114.90E; 32.33 N, 115.11E), Henan Province, in May 2013. The ticks were collected by dragging over the vegetation layer during daytime. Morphologic features were used to identify the species and developmental stage of ticks by an entomologist (Sun Y) [16]. 10–15 *H. longicornis* from each site were randomly selected and totally 144 ticks were included in our study. The predominant tick species was confirmed to be *H. longicornis*. Live *H. longicornis* ticks were subsequently sterilised in 75% ethanol and then washed up with deionised water for 5 min each to remove environmental contaminants. DNA and RNA were extracted from single tick using TIANamp DNA/RNA extraction kit (Tiangen, Beijing, China) according to the manufacturer’s instructions.

### Library preparation for high-throughput sequencing

The DNA/RNA extracted from 144 ticks were pooled respectively as the templates for library preparation. For prokaryotic pathogen screening, the pooled DNA was amplified in 50 μl reactions: 26.5 μl pure water, 10 μl 5 × phusion HF buffer (Thermo scientific, Hudson, NH, USA), 1 μl 10 mmol/L dNTPs (Thermo scientific), 2 μl of the 16S F and 16S R primers [17] (Invitrogen Corp., Carlsbad, CA), 0.5 μl Phusion High-Fidelity DNA polymerase (Thermo scientific), and 10 μl DNA(≈ 50 ng). The amplification was conducted according to a protocol involving initial denaturation for 30s at 98 °C and 35 cycles of 98 °C for 10 s, 55 °C for 30 s, 72 °C for 1 min, followed by a final extension at 72 °C for 7 min. Agarose gel with target fragments was purified using TIANgel Midi Purification Kit (Tiangen, Beijing, China) according to the manufacturer’s instruction. For eukaryotic pathogen screening, the pooled DNA was amplified as previously described using primers BTH-1F: cctgmgaracggctaccacatct and BTH-R: ttgcgaccatactccccca [18]. For virus screening, the pooled RNA was reverse transcripted with random hexmers to cDNA using Thermo Scientific Revert Aid First Strand cDNA Synthesis Kit (Thermo, Waltham, USA). The purified 16S and 18S PCR product, cDNA from RNA and DNA were sequenced with Ion Personal Genome Machine (PGM) System as described by Vogel and others [19]. Quality of the library was analysed using the Agilent 2100 Bioanalyzer (Agilent Technologies, Palo Alto, CA).

### Bioinformatics analysis

After sequencing, the individual sequence reads were filtered within the PGM software to remove low quality sequences. Sequences matching the PGM 3′adaptor were also automatically trimmed. Sequences that were shorter than 100 bp were deleted with an in-house python script. Each clean read was compared against the NCBI nucleotide collection (non-redundant nt database) using Blastn with default parameters (−v 5, −b 5, −w 35). The hit with the highest “Max Score” for every query was picked up, and the resulting hits were grouped by species according to its GI number. The number of reads and the total matched length of each species were calculated.

### Specific PCR for detection of three pathogens in ticks

Based on the results from the alignments, specific PCR was performed to confirm the presence of pathogens in the DNA and RNA of individual ticks. The genes used for phylogenetic analysis were as below: 16S rDNA for *Anaplasma* spp., *gltA* gene for *Rickettsia* spp., 18S rDNA for *Babesia* spp. and the S segment for SFTSV. Total RNA from each sample (0.1–1 μg) was used for reverse transcription using the SuperScript III First-Strand Synthesis System (Invitrogen). DNA extracted from each sample and cDNA reverse-transcripted from each sample were used as PCR template.

The targeted genes were amplified from template in 30 μl PCR mixtures containing 120 mmol/L of each primer (Table 1) [20–24], 60 mmol/L of each dNTP, 3 μl of 10 × r*Taq* PCR buffer (Takara, Dalian, China), and 1.5 U of r*Taq* DNA polymerase (Takara, Dalian, China). Amplification cycling conditions were as follows: denaturation for 3 min at 94 °C and 35 cycles of 94 °C for 45 s, 56 °C for 35 s, 72 °C for 1 min, followed by a final extension at 72 °C for 15 min. Amplified products were visualized with SYBR® Safe (Thermo, Waltham, USA) after electrophoresis in 2% agarose gel. For nested PCR, the second round were performed using the same reaction and cycling conditions as described above, and 1 μl of the first-round PCR production were used as template.

**Table 1:** Primers used in this study

Target	Gene	Primer	Primer name
*Anaplasma*	16S rRNA	Out1	TTGAGAGTTTGATCCTGGCTCAGAACG
		Out2(21)	CACCTCTACACTAGGAATTCCGCTATC
		Out2F	GATAGCGGAATTCCTAGTGTAGAGGTG
		317Pan(20)	AAAGGAGGTAATCCAGC
*Rickettsia*	gltA	CS2d	ATGACCAATGAAAATAATAAT
		CSEndr	CTTATACTCTCTATGTACA
		RpCS877F	GGGGACCTGCTCACGGCGG
		RpCS1258R(20)	ATTGCAAAAAGTACAGTGAACA
*Coxiella*	transposase	Cox_trans-3	GTAACGATGCGCAGGCGAT
		Cox_trans-4(23)	CCACCGCTTCGCTCGCTA
*Babesia*	18S rDNA	Piro0F	GCCAGTAGTCATATGCTTGTGTTA
		Piro1F	CCATGCATGTCTWAGTAYAARCTTTTA
		Piro5.5R	CCTYTAAGTGATAAGGTTCACAAAACTT
		Piro6R(22)	CTCCTTCCTYTAAGTGATAAGGTTCAC
SFTSV	S segment	BNYS1-F	TCTTCTCCATCAAGAACAGC
		BNYS1-R(24)	TTCGACAAAATTAGACCTCC

The primers for amplification in this study are presented in Table 1. The PCR ampliconswere directly sequenced using an ABI 3730 machine (Applied Biosystems, Foster City, CA, USA). To reveal the evolutionary status of identified pathogens of interest, the phylogenetic analysis was performed using the Mega 5.0 software (http://www.megasoftware.net). The alignment was made under default parameters. Phylogenetic analysis was performed by the Maximum Likelihood method. All positions containing alignment gaps and missing data were deleted (complete-deletion).

## Results

### Taxonomic classification

**Fig. 1:**
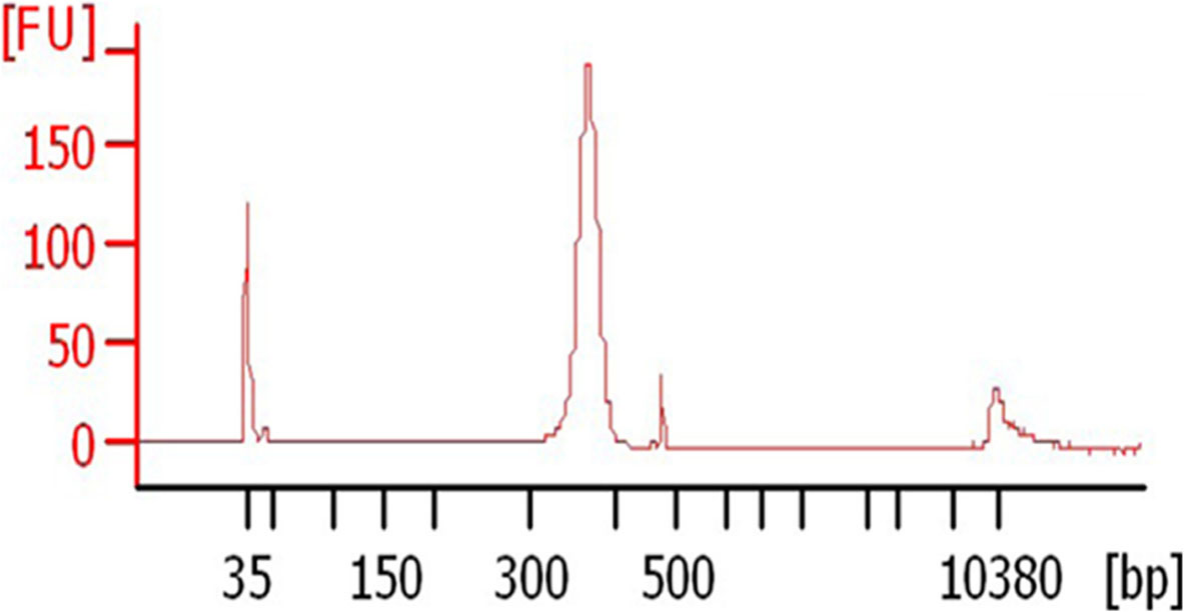
The fragments’ distribution of sequencing library. Quality of the libraries were analysed using the Agilent 2100 Bioanalyzer. The fragments length of the constructed library mainly ranged between 300 and 400 bp

**Fig. 2:**
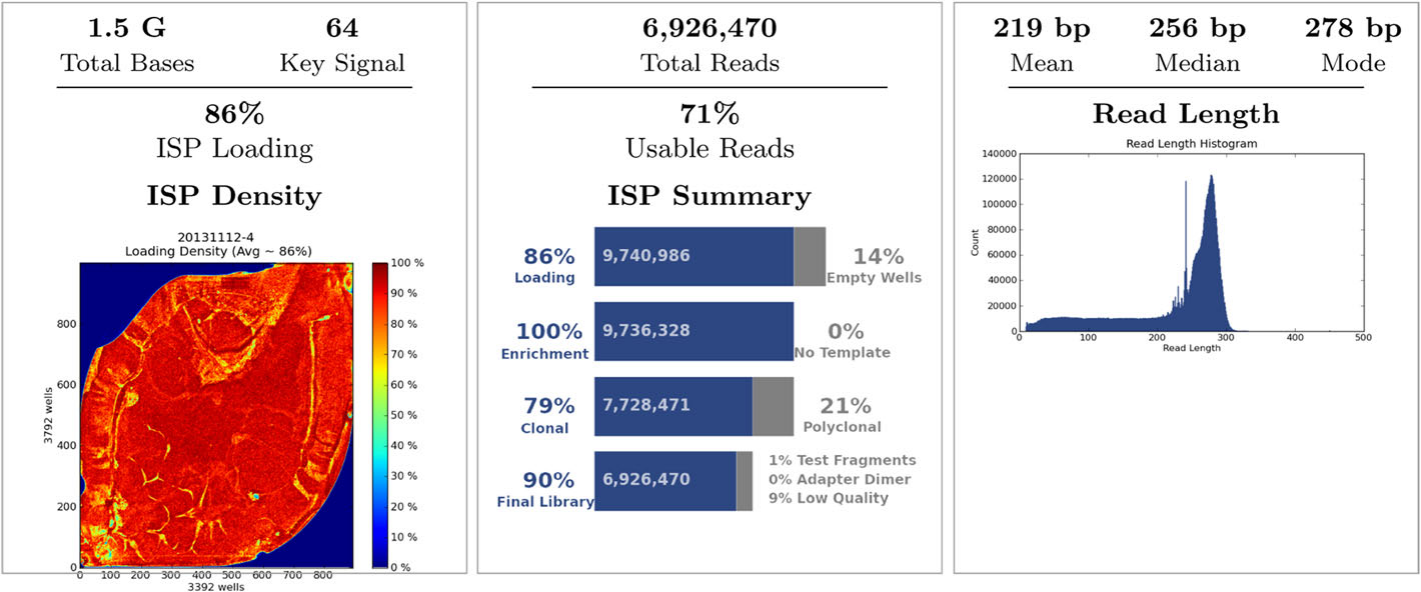
Run Report. Pseudo-colour image showing percent loading across the physical surface. This sequencing run had a 86% loading, which ensures a high ISP density. The number of total reads was up to 6 926 470 and 71% of the reads were usable. The median read length was 256 bp

The DNA and RNA extraction from a total of 144 adult *H. longicornis* ticks were respectively pooled into one sample, and subject to the high throughput sequencing. The fragments length of the constructed library ranged between 300 to 400 bp (Fig. 1). Totally 826 561 kb data were obtained, while 706 862 kb were of high quality (>Q20). A total of 6 926 470 reads were obtained with mean length of 233 bp (Fig. 2). The assignment of unassembled sequence reads to taxa based on BLASTN results revealed the existence of several pathogens, including *Anaplasma* spp., *Rickettsia* spp., *Babesia* spp., as well as SFTSV (Table 2).

**Table 2:** Potential pathogens presented by high-throughput sequencing

Species	Superkingdom	Total length^a^	Total number^b^
*Anaplasma bovis*	Prokaryota	38 461	157
*Anaplasma ovis*	Prokaryota	21 710	85
*Anaplasma phagocytophilum*	Prokaryota	2123	12
*Babesia bigemina*	Eukaryota	2225	19
*Babesia bovis*	Eukaryota	1188	15
*Babesia canis*	Eukaryota	22 734	199
*Babesia* cf. *divergens*	Eukaryota	2808	20
*Babesia divergens*	Eukaryota	24 651	95
*Babesia felis*	Eukaryota	4036	16
*Babesia gibsoni*	Eukaryota	15 349	69
*Babesia microti*	Eukaryota	29 114	307
*Babesia occultans*	Eukaryota	31 359	233
*Babesia odocoilei*	Eukaryota	14 817	155
*Babesia ovata*	Eukaryota	4636	22
*Babesia rossi*	Eukaryota	2239	22
*Babesia* sp. 28	Eukaryota	8543	71
*Babesia* sp. 4 NAN-2012	Eukaryota	2 054 498	12 132
*Babesia* sp. giraffe 1505	Eukaryota	8671	37
*Babesia* sp. Kh-Hj143	Eukaryota	68 397	241
*Babesia* sp. MA#230	Eukaryota	58 780	236
*Babesia* sp. MA#361–2	Eukaryota	56 703	252
*Babesia* sp. NJ5	Eukaryota	3230	21
*Babesia* sp. NV-1	Eukaryota	4606	34
*Babesia* sp. RWF-2013	Eukaryota	15 721	80
*Babesia* sp. sable antelope/2005	Eukaryota	8071	33
*Babesia* sp. SAP#091	Eukaryota	497 797	1919
*Babesia* sp. SAP#131	Eukaryota	177 543	813
*Babesia* sp. UR1	Eukaryota	5355	34
*Babesia* sp. ‘venatorum’	Eukaryota	9688	59
*Babesia* sp. YZ-2012	Eukaryota	3 803 579	15 839
*Coxiella burnetii*	Prokaryota	3 480 573	13 807
*Rickettsia australis*	Prokaryota	25 868	93
*Rickettsia conorii*	Prokaryota	9580	45
*Rickettsia heilongjiangensis*	Prokaryota	628 174	2387
*Rickettsia heilongjiangii*	Prokaryota	42 045	189
*Rickettsia japonica*	Prokaryota	5718	23
*Rickettsia prowazekii*	Prokaryota	5166	26
*Rickettsia rhipicephali*	Prokaryota	3309	14
*Rickettsia rickettsii*	Prokaryota	376 004	1519
*Rickettsia slovaca*	Prokaryota	2357	16
*Rickettsia* sp. BJ-90	Prokaryota	9265	38
*Rickettsia* sp. MSeoKT1	Prokaryota	2799	18
*Rickettsia* sp. T170-B	Prokaryota	1 056 955	4112
*Rickettsiella grylli*	Prokaryota	15 798	91
Severe fever with thrombocytopenia syndrome virus	Viruses	1420	5

^a^Total length: Sum of the reads that classified with the corresponding species

^b^Total number: Number of reads that classified with the corresponding species

### Confirmation of the pathogens and phylogenetic analysis

The remaining DNA/RNA from the 144 *H. longicornis* ticks were individually detected for the presence of these pathogens by PCR or RT-PCR (Additional file 2: Figure S1). The sequence analysis revealed the existence of *Anaplasma bovis* (Ehrlichia bovis, 13/144, 9.03%), *Anaplasma centrale* (2/144, 1.39%), *Rickettsia heilongjiangensis* (3/144, 2.08%), Rickettsia sp. LON-13 (1/144, 0.69%), *Rickettsia raoultii* (5/144, 3.47%), *Babesia* sp. (0.69%, 1/144) (Table 3). Three ticks were co-infected with SFTSV and *A. bovis.*

**Table 3:** Comparison of DNA sequence similarities between pathogens detected in ticks and the number of infection

Pathogen Genbank Match-Accession Number	Gene (Length)	Identity	Number of Infection (%)
*Ehrlichia bovis*-JN558824	16S (1490 bp)	99%	13 (9.03%)
*Anaplasma centrale*-AF283007	16S (1490 bp)	100%	2 (1.39%)
*Rickettsia heilongjiangensis*-EU665234	gltA (341 bp)	100%	3 (2.08%)
*Rickettsia* sp. LON-13-AB516964	gltA (341 bp)	100%	1 (0.69%)
*Rickettsia raoultii*	gltA (341 bp)	100%	5 (3.47%)
*Babesia* sp. MA#361–1-AB251610	18S (1619bp)	99%	1 (0.69%)
SFTSV-KC292288	S (491 bp)	100%	27 (18.75%)

**Fig. 3:**
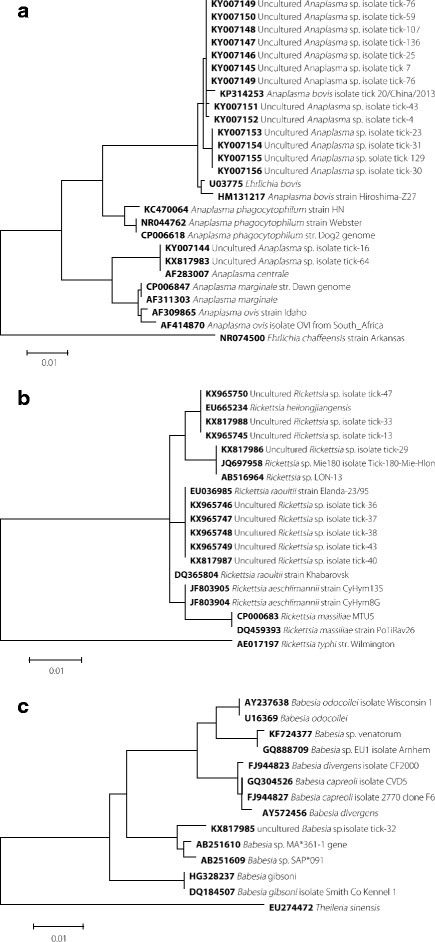
Phylogenetic analysis of confirmed pathogens of interests. Sequences were aligned using the MEGA5 (Version5.1) software package. Phylogenetic analysis was performed by the Maximum Likelihood method. All positions containing alignment gaps and missing data were deleted (complete-deletion). Bars indicate the percentage of sequence divergence. All positions containing alignment gaps and missing data were deleted (Complete-deletion). **a** Phylogenetic tree of bacteria belonging to *Anaplasma,* inferred from comparison of the partial 16S rDNA gene sequences. **b** Phylogenetic tree of bacteria belonging to *Rickettsia,* inferred from comparison of the partial gltA gene sequences. **c** Phylogenetic tree of bacteria belonging to *Babesia,* inferred from comparison of the partial 18S rDNA gene sequences. **d** Phylogenetic tree of bacteria belonging to SFTSV*,* inferred from comparison of the partial S segment

By phylogenetic analysis, the *Anaplasma* spp. (KY007144-KY007156, KX817983, KX817984) identified in the *H. longicornis* ticks were shown to be clustered with *A. centrale* and *A. bovis* (Fig. 3a). *Rickettsia*, which is recognized as medically important arthropod-vectored pathogens, was found involved in symbiosis within 9 *H. longicornis* ticks, and clustered into three different branchs (KX817986-KX817988, KX965745- KX96575), *R. heilongjiangensis*, *R. raoultii*, and *Rickettsia* sp. LON-13 (Fig. 3b). One of the *H. longicornis* ticks was found carrying *Babesia* sp. (GenBank No. KX817985). The phylogenetic analysis showed the 18S rRNA gene was most close to *Babesia* sp. MA*361–1 (GenBank No.AB251610) and *Babesia* sp. SAP*091(GenBank No.AB251609) (Fig. 3c). The represented SFTSV sequences (KX817989, KX965742-KX965744) obtained from four *H. longicornis* ticks were clustered with sequences obtained from local SFTS patients [25] (Fig. 3d).

## Discussion

In recent years, due to intense research interest in SFTS, there has been an increasing number of pathogens that were detected from *H. longicornis* ticks in SFTS endemic region [26]. Our study, based on the Next Generation Sequencing (NGS) methods, provided a broadened list of the microorganism harboured by this tick species, including prokaryotic and eukaryotic pathogens and viruses. In addition to SFTSV, other tick-borne pathogens, *A. bovis* (13/144, 9.03%), *A. centrale* (2/144, 1.39%), *R. heilongjiangensis* (3/144, 2.08%), *R. raoultii* (5/144, 3.47%), were detected as well.

Members of the genus *Anaplasma* include *A. phagocytophilum*, *A.marginale*, *A. bovis*, *A. ovis*, *A. platys* and *A. centrale*, all of which are obligate intracellular bacteria that infect a variety of cell types [27]. In China, wild and domestic ruminants play active roles as *Anaplasma*.sppcarriers and reservoirs. The presence of *A. bovis* in sheep and goats has been reported in Northwest, Central and Southern China [28, 29]. *A. centrale* has been reported in goats and sheep from South-eastern China [30]. Our results indicate the epidemic of *A. bovis* and *A. centrale* in central China. In the study, spotted fever group *Rickettsia*, including *R. heilongjiangensis*, *R. raoultii* and *Rickettsia* sp. LON-13 was detected in *H. longicornis* ticks in the same region. *R. heilongjiangensis* can cause spotted fever in humans, which was detected in *Dermacentor. silvarum and H. longicornis* ticks in Heilongjiang Province and Zhejiang Province [31–33]*. R. raoultii,* the predominant *Rickettsia* found in *Dermacentor silvarum* ticks in China-Russia border areas [34], was also found in this area. According to phylogenetic analysis, *Rickettsia* sp. LON-13 was clustered in spotted fever group, and further investigation should be taken in livestock and human. *Babesia* sp. was detected in only one tick. According to phylogenetic analysis of 18S rRNA gene, *Babesia* from this tick was clustered between *Babesia divergens* and *Babesia gibsoni*, closing to *Babesia* sp. MA*361–1. This finding indicates the possible existence of a new *Babesia* species in local environment. Its prevalence in livestock and the risk to human beings requires to be investigated by an enhanced surveillance in the future.

Co-infection of *A.bovis* with SFTSV was identified, which is interesting while unexpected. Before the discovery of SFTSV, co-infection between *Babesia microti* (KJ715163) and *Rickettsia* sp. (KJ715194), *Theileria luwenshuni* (KJ715167) and *Ehrlichia* sp*.* (KJ715196), *T. luwenshuni* (KJ715168) and *Anaplasma phagocytophilum* (KJ715199) in *H. longicornis* ticks had been reported [26]. In addition, dual infection with *A. phagocytophilum* and *B. microti* in a *Rattus norvegicus* was found in this region [35]. However, the evidence of human dual infection was only recently reported from our previous research on the existence of CRT (*Candidatus Rickettsia tarasevichiae*) infection in clinical diagnosed SFTS patients [36]. Taken all of the findings together, we propose that in SFTS endemic areas bacterial infection including *Anaplasma* spp., *Rickettsiae* spp., *Babesia* spp., and co-infecitons of various tick-borne pathogens should be considered for patients after tick bite.

Since the discovery of SFTS in China, enormous efforts have been applied to identify SFTSV infection in both human being and the predominant tick species. However, other tick-borne pathogens were largely neglected. Due to nonspecific clinical presentation and less access to confirmatory laboratory findings, it is rather difficult to make diagnosis. In addition, novel *Rickettsia* and *Babesia* species of undetermined pathogenicity continue to be detected from ticks, highly possible to cause human illness. The current findings might have important application in determining the etiological determination in SFTS endemic region with *H. longicornis* as the predominant tick species, the most important tick-borne infectious disease not only in China, but also in countries where SFTSV infection has been reported.

## Conclusions

In the study, a broadened list of the microorganism harboured by *H. longicornis* was provided. In SFTS region with abundant *H. longicornis*, prokaryotic infection including *Anaplasma* spp., *Rickettsiae* spp., and *Babesia* spp. should also be considered. The possibility of their co-infection with tick-borne viral pathogens in *H. longicornis* ticks, and dual infection in human, should be acknowledged by the clinicians. Specially, a novel *Babesia* species was identified in local natural foci, which needs further investigation in the future.

## Additional files

Additional file 1: Multilingual abstracts in the five official working languages of the United Nations. (PDF 532 kb)

Additional file 2: **Figure S1.** Experimental confirmation of predicted pathogens of interest predicted by bioinformatics. A), PCR amplification of the *H. longicornis* ticks to confirm the predicted *Anaplasma* spp.. S1 represented PCR amplification with primer Out1, Out2; S2 represented PCR amplification with primer Out2F, 317Pan. B), The second run of nested PCR (with primer RpCS877F, RpCS1258R) amplification of *H.longicornis* ticks to confirm the predicted *Rickettsia* spp.. C), The second run of nested PCR (with primer Piro1F, Piro5.5R) amplification of *H. longicornis* ticks to confirm the predicted *Babesia* spp.. D), The PCR (with primer BNYS1-F, BNYS1-R) amplification of the *H. longicornis* ticks to confirm the predicted SFTSV. (JPEG 80 kb)

## Abbreviations

SFTS: Severe fever with thrombocytopenia syndrome
SFTSV: Severe fever with thrombocytopenia syndrome virus
