# Supplementary material for: Identification of tick-borne pathogen diversity by metagenomic analysis in Haemaphysalis longicornis from Xinyang, China
Source: Infect Dis Poverty. 2018 May 7;7:45. doi: 10.1186/s40249-018-0417-4 (PMC5937033; doi:10.1186/s40249-018-0417-4)
Supplement: Supplementary file 1 — Multilingual abstracts in the five official working languages of the United Nations. (PDF 532 kb) [file 40249_2018_417_MOESM1_ESM.pdf]

Translation of the abstract into the five official working languages of the United Nations

لو تشوانغ، خوان دو، شياو-مينغ تسوى، هاو لى، فانغ تانغ، بان-هي تشانغ، جيان-قونغ هو، يى-قانغ تونغ، تشى-تشون فنغ، وى ليو

Lu Zhuang, Juan Du, Xiao-Ming Cui, Hao Li, Fang Tang, Pan-He Zhang, Jian-Gong Hu, Yi-Gang Tong, Zhi-Chun Feng, Wei Liu

ملخص

خلفية: قراد القرموشة طويلة القرون Haemaphysalis longicornis هو ناقل مهم يمكن أن يحمل وينقل مجموعة واسعة من العوامل المسببة للأمراض. الهدف من هذه الدراسة هو فحص مجموعة متنوعة من العوامل المسببة للأمراض التي يحملها قراد القرموشة طويلة القرون Haemaphysalis longicornis في منطقة واحدة موبوءة بأمراض منقولة بالقراد، وتقدير خطر الإصابة البشرية التي تشكلها لدغات القراد.

الطريقة/الأسلوب: تم جمع القراد البالغة/الكبيرة (والتي تسلك طريقة تسمى" questing " للبحث عن مضيفها) في شينيانغ - وسط الصين. تم استخراج الحمض النووي الريبي منزوع الاكسجين DNA والحمض النووي الريبي RNA من 144 قراد (قرموشة طويلة القرون Haemaphysalis longicornis) بشكل فردي، وتجميعها على التوالي في عينة واحدة لإجراء تسلسل عالي السرعة high-throughput sequencing. تمت مقارنة قراءات نظيفة مع قاعدة بيانات المركز الوطني لمعلومات التقنيات الحيوية NCBI الخاصة بتجميع النيوكليوتيدات وتم إجراء تفاعل محدد للبوليميراز المتسلسل PCR لتأكيد وجود العامل المسبب للمرض. تم إجراء تحليل النشوء والتطور لاستكشاف الحالة التطورية للعوامل المسببة للأمراض.

النتائج: كشفت إحالة القراءات الى الأصانيف (جمع أصنوفة/وحدة تصنيفية) المستندة على نتائج BLASTN عن وجود عدة مسببات محتملة للأمراض، بما في ذلك أنواع أنابلاسما، أنواع ريكيتسيا، أنواع بابيسيا، فضلا عن حمى شديدة ينتجها فيروس بونيا المسبب لمتلازمة نقص الصفيحات الدموية (SFTSV). كشفت تفاعلات تفاعل البوليميراز المتسلسل عن وجود أنابلاسما بوفيس (144/13، 9.03 %)، أنابلاسما سينترال (144/2، 1.39 %)، ريكيتسيا هيلونغجيانغنسيس (144/3، 2.08 %)، أنواع ريكيتسيا LON-13 (144/1، 0.69 %)، ريكيتسيا راولتي (144/5، 3.47 %)، أنواع بابيسيا (144/1، 0.69 %). وكان فيروس بونيا المسبب لمتلازمة نقص الصفيحات الدموية (SFTSV) العامل الممرض الأكثر اكتشافا/ضبطا مع معدل إيجابي قدره 18.75 % (144/27). ثلاثة من القراد (2.08 %) كانت مصابة بعاملين ممرضين معا (فيروس بونيا المسبب لمتلازمة نقص الصفيحات الدموية (SFTSV) وأنابلاسما بوفيس).

الاستنتاجات: قدمت دراستنا قائمة موسعة من الكائنات الحية الدقيقة التي يؤويها قراد (قرموشة طويلة القرون Haemaphysalis longicornis). في المناطق الموبوءة الغير معروفة سابقا، ينبغي الأخذ بعين الاعتبار (وجود) عدوى سببها بدائية النواة وحقيقية النواة بما في ذلك أنواع أنابلاسما، أنواع ريكيتسيا، أنواع بابيسيا، جنبا إلى جنب مع الفيروس المعروف بونيا المسبب لمتلازمة نقص الصفيحات الدموية (SFTSV) عند المرضى الذين تعرضوا سابقا للدغة القراد. تم التعرف على أنواع جديدة من البابيسيا في بؤر طبيعية محلية، وتحتاج إلى مزيد من البحث في المستقبل.

Translated from English version into Arabic by doctorammar, through

TRANSLATORS
WITHOUT BORDERS

通过宏基因组分析中国信阳长角血蜱中蜱传病原体多样性

Lu Zhuang, Juan Du, Xiao-Ming Cui, Hao Li, Fang Tang, Pan-He Zhang, Jian-Gong Hu, Yi-Gang Tong, Zhi-Chun Feng, Wei Liu

引言：长角血蜱（*Haemaphysalis longicornis*）可以携带和传播多种病原体。本研究的目的是对蜱媒传染病流行地区长角血蜱携带的病原体展开全面的筛查，并评估蜱叮咬人引起的感染风险。
方法：在中国中部的河南省信阳地区收集成蜱。从144只长角血蜱提取基因组DNA和RNA，作为模板建立高通量测序文库。原始数据经过初步处理，与NCBI nt数据库进行比较后，根据病原体预测结果，以每一只蜱的DNA和RNA作为模板进行特异PCR扩增，确认病原体的存在，并进行系统发育分析，了解病原体间的进化关系。
结果：BLASTN序列比对结果提示样本中可能存在以下几种病原体：无形体属、立克次氏体属、巴贝斯虫属，以及发热伴血小板减少综合征布尼亚病毒（SFTSV）。 特异PCR验证了这些病原体的存在，包括牛无形体（*Anaplasma bovis*，13/144，9.03%），中央无形体（*Anaplasma centrale*, 2/144，1.39％），黑龙江立克次体（*Rickettsia heilongjiangensis*， 3/144，2.08％），*Rickettsia* sp. LON-13（1/144，0.69%），拉欧蒂立克次体（*Rickettsia raoultii*，5/144，3.47％），巴贝斯虫（*Babesia* sp. 1/144， 0.69%）。 其中，SFTSV检出率最高，为18.75％（27/144）。其中3个蜱（2.08％）检测出SFTSV和牛无形体双重感染。
结论：研究对长角血蜱携带的微生物进行了全面的筛查。除新布尼亚病毒外，该地区蜱咬患者也应该考虑原核生物和真核生物感染，包括无形体，立克次氏体，巴贝斯虫。同时，我们的结果提示在当地的自然疫源地中存在一种新的巴贝斯虫，需要进一步研究。

Translated from English version into Chinese by Lu Zhuang

Identification de la diversité pathogène transmise par les tiques par analyse métagénomique *Haemaphysalis longicornis* de Xinyang, Chine

Lu Zhuang, Juan Du, Ciao Xiao-Ming, Hao Li, Fang Tang, Zhang Pan-He, Jian-Gong Hu, Tong Yi-Gang, Zhi-Chun Feng, Wei Liu

Résumé

Contexte: *Haemaphysalis longicornis* est un vecteur important qui peut transporter et transmettre une grande variété de pathogènes. Le but de cette étude est d'examiner la variété des pathogènes portés par *Haemaphysalis longicornis* dans une zone d'épidémie de maladies transmises par les tiques, et évaluer le risque d'infection humaine posé par les piqûres de tiques.
Méthodes: Des tiques adultes ont été collectées à Xinyang en Chine centrale. L'ADN et l'ARN ont été extraits de 144 tiques *H. longicornis* individuellement et regrouper en un seul échantillon pour un séquençage à haut débit. Des lectures propres ont été comparées à la base de données de la collection de nucléotides du NCBI et une PCR spécifique a été réalisée pour confirmer la présence d'agent pathogène. L'analyse phylogénétique a été réalisée pour étudier le statut évolutif des pathogènes.
Résultats: L'attribution de lectures à des taxons basés sur les résultats BLASTN a révélé l'existence de plusieurs pathogènes potentiels, y compris *Anaplasma* spp., *Rickettsia* spp., *Babesia* sp., ainsi qu'une forte fièvre avec syndrome de la thrombocytopénie bunyavirus (SFTSV). La PCR confirmative a révélé l'existence de *Anaplasma bovis* (13/144, 9,03%), *Anaplasma centrale* (2/144, 1,39%), *Rickettsia heilongjiangensis* (3/144, 2,08%), *Rickettsia* sp. LON-13 (1/144, 0,69%), *Rickettsia raoultii* (5/144, 3,47%), *Babesia* sp. (0,69%, 1/144). Le SFTSV était l'agent pathogène le

plus fréquemment détecté avec un taux positif de 18,75% (27/144). Trois des tiques (2,08%) étaient co-infectées par le SFTSV et *Anaplasma. bovis.*

Conclusions: Notre étude a fourni une liste élargie de micro-organismes hébergés par *H. longicornis*. Dans les régions endémiques jusque-là non reconnues, les infections procaryotes et eucaryotes *Anaplasma* spp., *Rickettsiae* spp., et *Babesia* spp. devraient être considérés, avec le SFTSV bien connu pour les patients ayant des antécédents de piqûres de tiques. Un espèce *Babesia* inédit a été identifiée dans des foyers naturels locaux, ce qui nécessite des recherches plus poussées à l'avenir.

Translated from English version into Russian by Abdourahamane, through

TRANSLATORS WITHOUT BORDERS

Использование метагеномного анализа для выявления разнообразия клещевых патогенов в *Haemaphysalis longicornis* в Синьяне, Китай

Лу Чжуан, Цзюань Ду, Сяо-Мин Цуй, Хао Ли, Фан Тан, Пань-Хэ Чжан, Цзянь-Гун Ху, И-Ган Тун, Чжи-Чунь Фэн, Вэй Лю

Аннотация

С
п
р
а
в
[illegible]
[illegible]
[illegible]
[illegible]
[illegible]
ы
и

[illegible] Результаты: [illegible] BLASTN было выявлено присутствие нескольких потенциальных патогенов, включая виды *анаплазмы (Anaplasma* spp.), риккетсий (*Rikettsia* spp.), бабезии (*Babesia* sp.), а также острую лихорадку с тромбоцитопеническим синдромом вируса bunyavirus (SFTSV). Подтверждающая полимеразная цепная реакция (ПЦР) выявила присутствие *Anaplasma bovis* риккетсии (*Rickettsia* sp). LON-13 (1/144; 0,69%), *Rickettsia raoultii* (5/144; 3,47%), бабезии уровне положительных результатов 18,75% (27/144). Три клеща (2,08%) были одновременно

и
я
н
ы

представляет собой важного переносчика, способного как нести на себе, так и передавать широкий спектр патогенов. Целью данного исследования является изучение разновидности патогенов, переносимых *Haemaphysalis longicornis* в одном районе эпидемий клещевых

заражены SFTSV и *Anaplasma. bovis.*
Выводы: Данное исследование расширило список микроорганизмов, средой обитания которых является *H. longicornis*. В районах, ранее не считавшихся эндемичными, при наличии у пациентов истории клещевых укусов, наряду с хорошо известными SFTSV, следует принимать во внимание прокариотические и эукариотические инфекции, включая виды анаплазм (*Anaplasma* spp.*)*, *риккетсий (Rickettsiae* spp), а также бабезии (*Babesia* spp.*)*. В местных природных очагах был выявлен новый вид бабезии (*Babesia* species), требующий дальнейшего углублённого изучения в будущем.

Translated from English version into Russian by Liudmila Tomanek, through

Identificación de diversidad de patógenos transmitidos por garrapatas a través de análisis de la metagenómica en *Haemaphysalis longicornis* de Xinyang, China

Lu Zhuang, Juan Du, Xiao-Ming Cui, Hao Li, Fang Tang, Pan-He Zhang, Jian-Gong Hu, Yi-Gang Tong, Zhi-Chun Feng, Wei Liu

Resumen
Contexto: *Haemaphysalis longicornis* es un vector importante que puede portar y transmitir una gran variedad de patógenos. La meta de este estudio es examinar la variedad de patógenos portados por *Haemaphysalis longicornis* en un área con epidemia de enfermedades transmitidas por garrapatas, y estimar el riesgo de infección humana que suponen las picaduras de garrapata.
Métodos: Se obtuvieron garrapatas adultas atrapadas en Xinyang en China central. Se extrajeron el DNA y RNA de 144 garrapatas *H. longicornis* individualmente, y se agruparon en una muestra para secuenciación de alto rendimiento. Los datos limpios se compararon con la base de datos de la colección de nucleótidos de NCBI, y se realizaron PCR específicos para confirmar la presencia de patógenos. Se realizó un análisis filogenético para explorar el estado evolucionario de los patógenos.
Resultados: La asignación de lecturas para los taxa basados en resultados BLASTN reveló la existencia de diversos patógenos potenciales, incluyendo *Anaplasma* spp., *Rickettsia* spp., *Babesia* sp., así como fiebre severa con síndrome trombocitopénico (SFTSV). Confirmación PCR reveló la existencia de *Anaplasma bovis* (13/144, 9.03%), *Anaplasma centrale* (2/144, 1.39%), *Rickettsia heilongjiangensis* (3/144, 2.08%), *Rickettsia* sp. LON-13 (1/144, 0.69%), *Rickettsia raoultii* (5/144, 3.47%), *Babesia* sp. (0.69%, 1/144). SFTSV fue el patógeno más frecuentemente detectado con una tasa positiva del 18.75% (27/144). Tres de las garrapatas (2.08%) estaban co-infectadas con SFTSV y *Anaplasma. bovis.*
Conclusiones: Nuestro estudio proporciona una lista ampliada de microorganismos albergados por *H. longicornis*. En regiones endémicas previamente no reconocidas, infecciones procariotas y eucariotas incluyendo *Anaplasma* spp., *Rickettsiae* spp., y *Babesia* spp. deben ser consideradas, al igual que el conocido SFTSV, en los pacientes con historia de picaduras de garrapata. Una especie

nueva de *Babesia* fue identificada en focos naturales locales, que requiere investigación adicional en el futuro.

T

l
a
t
e
d

f
r
o
m

E
n
g
l
i
s
h

v
e
r
s
i
o
n

i
n
t
o

R
u
s
s
i
a
n

b
