# Supplementary figures and images for: Identification of tick-borne pathogen diversity by metagenomic analysis in Haemaphysalis longicornis from Xinyang, China
Source: Infect Dis Poverty. 2018 May 7;7:45. doi: 10.1186/s40249-018-0417-4 (PMC5937033; doi:10.1186/s40249-018-0417-4)

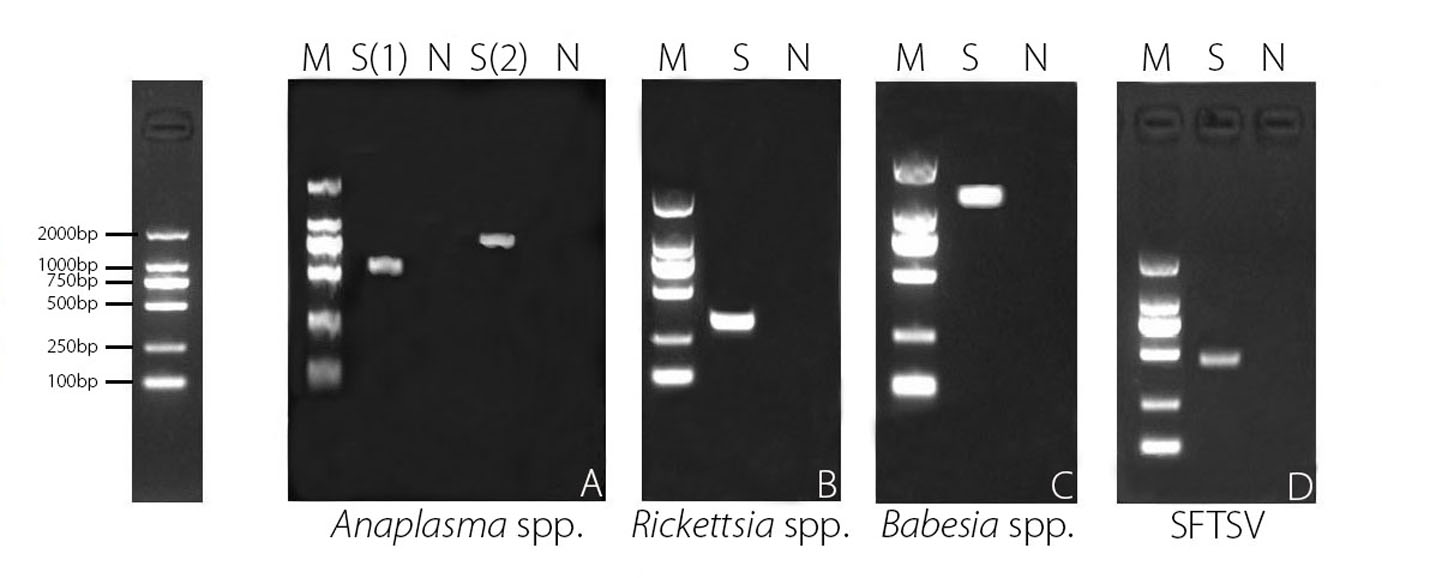

Supplement: Supplementary file 2 — Figure S1. Experimental confirmation of predicted pathogens of interest predicted by bioinformatics. A), PCR amplification of the H. longicornis ticks to confirm the predicted Anaplasma spp.. S1 represented PCR amplification with primer Out1, Out2; S2 represented PCR amplification with primer Out2F, 317Pan. B), The second run of nested PCR (with primer RpCS877F, RpCS1258R) amplification of H.longicornis ticks to confirm the predicted Rickettsia spp.. C), The second run of nested PCR (with primer Piro1F, Piro5.5R) amplification of H. longicornis ticks to confirm the predicted Babesia spp.. D), The PCR (with primer BNYS1-F, BNYS1-R) amplification of the H. longicornis ticks to confirm the predicted SFTSV. (JPEG 80 kb) [file 40249_2018_417_MOESM2_ESM.jpg]
